# Supplementary material for: MetaRibo-Seq measures translation in microbiomes
Source: Nat Commun. 2020 Jun 29;11:3268. doi: 10.1038/s41467-020-17081-z (PMC7324362; doi:10.1038/s41467-020-17081-z)
Supplement: Supplementary file 10 — Supplementary Data 7 [file 41467_2020_17081_MOESM10_ESM.zip › File2/Confidence_High_Taxonomy/260744_out.krona.html]

Javascript must be enabled to view this page.

members
magnitude
magnitudeUnassigned
count
unassigned
taxon
rank

260744\_out

13

2
13
superkingdom

1239
12
phylum

12
909932
class

order
1843489
12

31977
12
family

12
29465
genus

species
12

SRS012969\_contig\_number\_36546SRS019496\_contig\_number\_contig-100\_3652.51378SRS019910\_contig\_number\_contig-100\_36.136218SRS054590\_contig\_number\_contig-100\_1.29010SRS063489\_contig\_number\_6419SRS065397\_contig\_number\_5961SRS076804\_contig\_number\_20852SRS1041091\_contig\_number\_10292SRS104311\_contig\_number\_22292SRS1055038\_contig\_number\_contig-100\_14990.14991SRS146813\_contig\_number\_7727SRS147614\_contig\_number\_5595
1262980

1
976
phylum

class
1
200643

order
171549
1

family
1
2005519

1
397864
genus

1
487174

SRS050752\_contig\_number\_19388
species
